# Unraveling the mechanism of fragrance release in *Cestrum nocturnum* through transcriptome and volatile compound profiling

**DOI:** 10.1038/s41598-025-99542-3

**Published:** 2025-05-02

**Authors:** Sitong Qiao, Anqi Ding, Tao Liu, Hangcheng Hu, Mengting Li, Jiyang Wang, Leixing Deng, Shiheng Lyu

**Affiliations:** 1 College of Geography and Planning, Chengdu University of Technology, No. 1 East Third Road Erxianqiao, Chenghua District, Chengdu, 610059 Sichuan China; 2 Research Center of National Park, Sichuan Key Research Base for Social Sciences, Chengdu, 610059 Sichuan China; 3 Human Geography Research Center of Qinghai-Tibet Plateau and Its Eastern Margin, Chengdu, 610059 Sichuan China; 4 Agricultural and Rural Bureau of TaoJiang County, Yiyang, 413000 Hunan China; 5 College of Horticultures, Fujian Agriculture and Forestry University, Fuzhou, 350000 Fujian China

**Keywords:** *Cestrum nocturnum*, Differential regulatory genes, Fragrance biosynthesis, Night-blooming, Phenylpropanoid biosynthesis, Molecular biology, Transcription, Transcriptomics

## Abstract

*Cestrum nocturnum* is a plant that blooms and emits fragrance at night but the mechanism behind its fragrance release remains poorly understood. Here, the floral substances and differential regulatory genes in the petals of *C. nocturnum* at night were explored by HS-SPME-GC/MS and transcriptome sequencing. Benzaldehyde, phenyl acetaldehyde and benzyl acetate were identified as the main floral scent substances involved in the phenylpropanoid biosynthesis pathway of *C. nocturnum*. The DEGs in this pathway, *PALs*, *PAAS*, *PARs*, *4CLs*, *CFATs*, *BEAT*, *EGSs* and *BAMT*, showed high expression levels during flowering. Additionally, these genes may play an important role in the formation of a regulatory network for floral scent substance synthesis. DEGs in day/night alternates and plant hormone signalling pathways also had important functions in flowering and fragrance release. These included *CO*, *FT* and genes related to the promotion of phytohormone (GA, ABA, IAA, BR) biosynthetic and signal transduction, which showed high expression levels during flowering. These results provide a theoretical reference for the expression levels of differentially expressed functional genes in the flowering and fragrance release of *C. nocturnum* and lay a foundation for further research on night-blooming aromatic flowers.

**Supplementary Information:**

The online version contains supplementary material available at 10.1038/s41598-025-99542-3.

## Introduction

Floral scent is an important attribute of plants. It is used to evaluate the quality of ornamental plants^1^. It attracts pollinators and plays an important role in plant growth, development and reproduction^2,3^. Floral scent is due to the release of volatile, low-molecular-weight compounds by plants^4,5^. Floral scent compounds are secondary metabolites in plants and can be classified into three major groups: phenylpropanoids, terpenoids and fatty acid derivatives^6,7^. In previous studies, the most reported metabolites were phenylpropanoids and terpenoids^8–10^.

The phenylpropanoid biosynthesis pathway begins with phenylalanine, which is transformed into cinnamic acid and phenylacetaldehyde via catalysis by phenylalanine ammonia lyase (*PAL*) and phenylacetaldehyde synthase (*PAAS*)^11^. The next reactions are catalysed by 4-coumarate-CoA ligase (*4CL*), phenylacetaldehyde reductase (*PAR*) and Cinnamate-4-hydroxylase (*C4H*) to produce cinnamoyl-CoA, phenylethyl alcohol and *p*-Coumaric acid, respectively^12–14^. cinnamoyl-CoA is a prerequisite for the synthesis of benzaldehyde^14^. *PAR* has been reported to catalyse the reduction of benzaldehyde to benzyl alcohol^15^. Benzyl acetate is produced through the catalytic action of benzyl alcohol acetyltransferase (*BEAT*)^3,16^. Benzaldehyde is catalyzed by benzaldehyde dehydrogenase (*BALDH*) to produce benzoic acid^17^. Benzoic acid carboxyl methyl transferase (*BAMT*) catalyses the transformation of benzoic acid into methyl benzoate^3,13,18^. *p*-Coumaric acid produces coniferyl alcohol through a series of enzymatic reactions^14^. Coniferyl acetate synthesis is due to the CFAT (coniferyl alcohol acetyltransferase)-catalysed reaction of coniferyl alcohol^19^. The synthesis of eugenol is catalysed by eugenol synthase (*EGS*) with coniferyl acetate as the substrate^20^. All of these reactions are part of the phenylpropanoid biosynthetic pathway. Terpenoids can be classified as hemiterpenes, monoterpenes, sesquiterpenes, diterpenes, triterpenes, tetraterpenes and polymorphic terpenes, depending on the number of isoprene structures^7,21^. The most common of these are monoterpenes and sesquiterpenes^8^. Linalool, chlorophyll and β-arylene are the most reported compounds in these two groups^4^.

Floral scent compounds vary greatly between plants. For example, phenethyl alcohol, benzyl benzoate and phenethyl benzoate are the main floral components of *Murraya paniculata*^22^. Ester and terpene constituents of *Dendrobium* Sw. account for more than 80% of all its floral scent compounds^23^. The main floral scent components of *Peturnia hybrida* are phenylpropanes, such as methyl benzoate, benzaldehyde, phenylethyl alcohol and phenylacetaldehyde, with small amounts of sesquiterpenes^24^.

The main floral scent components of *Camellia japonica* in blooming flowers are phenethyl alcohol and methyl salicylate, *CaPAR and CaSAMT* are critical for the synthesis of floral aroma substances^25^. *PAAS*, *BPBT* and *PAL* are key genes for the synthesis of floral scent substances in *Petunia hybrida*^26^. However, studies on the molecular mechanisms of night-blooming aromatic flowers are not well understood.

The process of releasing fragrance accompanies plant flowering. *CONSTANS* (*CO*) and *flowering locus T* (*FT*) are two important genes in the flowering regulatory network that directly regulate flowering time in plants^27–29^. *CO* promotes *FT* transcription under long-day conditions in *Arabidopsis thaliana,* with overexpression of the *CO* gene elevating *FT* expression levels^27^. When *FT* is overexpressed in *Chrysanthemum indicum*, it can accelerate entry into its early reproductive stage^30^. The overexpression of *CiFT* in *Poncirus trifoliata* leads to a significant advance in flowering time^31^. Additionally, the hormones included gibberellic acid (GA), abscisic acid (ABA), brassinosteroid (BR) and indole-3-acetic acid (IAA), which have important roles in the flowering process^32–34^.

*Cestrum nocturnum* L. belongs to the genus *Cestrum* in the *Solanaceae*, a typical long-short-day plant widely grown in southern China^2^. It is an evergreen shrub 2–3 m tall with slender branches and elegant morphology^35^. In contrast to traditional plants, it is a night-blooming aromatic plant^36^; the petals of *C. nocturnum* are closed during the day but open at night and release a strong fragrance. Its petal movement and fragrance release patterns are similar to other plants, such as *Epiphyllum oxypetalum*^37^ and *Oenothera biennis*^38^. A variety of volatile compounds, including linalool, benzaldehyde, benzyl alcohol, phenyl acetaldehyde, *cis*-jasmone, and benzyl acetate, were identified in *C. nocturnum*^36^. However, the molecular mechanisms underlying night flowering and aroma release are poorly understood.

In this study, petals of *C. nocturnum* were collected at different times of the night. They were selected for the exploration of floral scent substances and differentially regulated genes by headspace solid-phase microextraction gas chromatography–mass spectrometry (HS-SPME-GC/MS) and transcriptome sequencing. The results provide a theoretical reference for the flowering and fragrance release mechanism of night-blooming aromatic plants.

## Materials and methods

### Plant materials

**Fig. 1:**
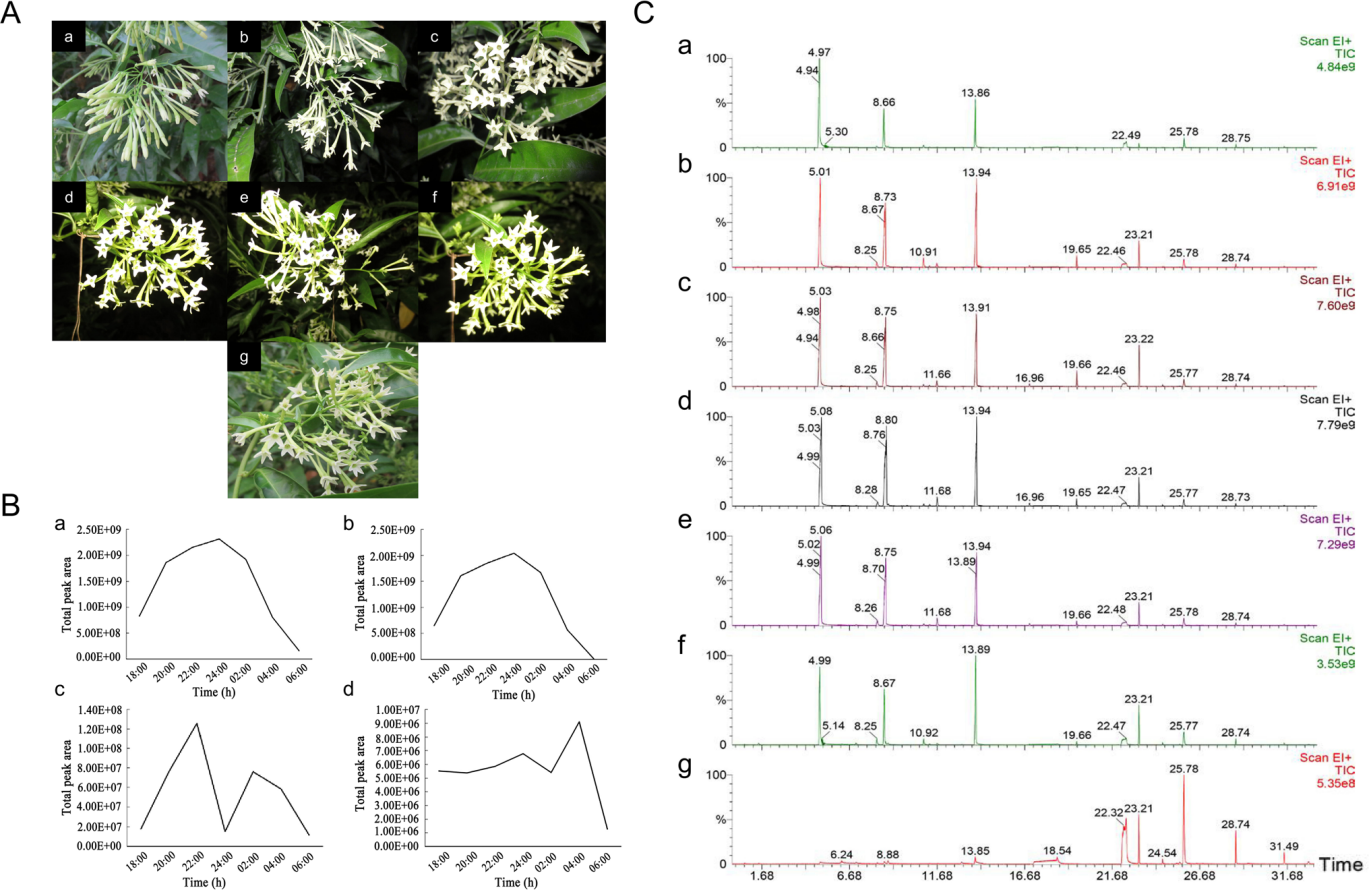
Flowering state at night and analysis of volatile components in petals of *C. nocturnum*. (**A**) Flowers at different times: (**a**) 18:00 (CK); (**b**) 20:00; (**c**) 22:00; (**d**) 24:00; (**e**) 02:00; (**f**) 04:00; (**g**) 06:00. (**B**) Peak area variation of volatiles: (**a**) total peak area; (**b**) phenylpropanoids; (**c**) terpenoids; (**d**) fatty acid derivatives. (**C**) Total ion chromatogram (TIC) of volatiles at different times: (**a**) 18:00 (CK); (**b**) 20:00; (**c**) 22:00; (**d**) 24:00; (**e**) 02:00; (**f**) 04:00; (**g**) 06:00.

Flowers of *C. nocturnum* were collected in August 2016 on the campus of Fujian Agriculture and Forestry University (E 119° 19′, N 26° 06′), China. Flowers were collected every 2 h from 18:00 to the next day at 06:00. The petal movement pattern has three consecutive phases: a gradual opening phase from 18:00 to 22:00, a full opening phase from 22:00 to the next day at 02:00, and a gradual closing phase from 02:00 to 06:00 (Fig. 1A). Petals collected at 18:00 were used as the control check (CK). Six flowers with similar growth conditions were collected each time, immediately put into headspace vials, pressed tightly with crimping tongs and subjected to headspace extraction. The same batches of materials were used for transcriptome and qRT-PCR experiments in 2017 and 2022, respectively.

### Extraction and analysis of volatile components

HS-SPME/GC–MS was used to collect and analyse volatile compounds^15^. Six intact flowers were sealed in a 20 mL extraction bottle and equilibrated at 25 °C for 60 min. A SPME fibre was used for the extraction and adsorption of volatile components over 30 min. The injection temperature was adjusted to 260 °C and a capillary column was used to separate the compounds using helium as the carrier gas at 1 mL min^−1^. The sample was injected in a split stream with a split ratio of 1:20. The GC oven temperature program was initiated at 50 °C with a 5-min hold, followed by a temperature ramp of 3.5 °C min^−1^ to 90 °C, and subsequently increased at a rate of 5 °C min^−1^ to 210 °C with a hold for 3 min.

The relative content of volatile components was calculated by peak area normalization. The peak area calculations and substance characterisation were performed using TurboMass v6.1.0 and the NIST11 search library with a default match value of ≥ 750 as the screening criterion. Among the detected substances, the one with the highest match value was selected as the test result and its Chinese name was obtained by searching according to the CAS number.

### Transcriptome sequencing and assembly

Total RNA was extracted from petal samples using the RNAprep Pure Plant Kit (DP441, Tiangen, China) according to the manufacturer’s protocol and then tested for integrity, purity and concentration. Samples of *C. nocturnum* collected at 18:00, 22:00, 02:00 and 06:00 were submitted to Biomarker Technologies for sequencing and assembly in three biological replicates experiments. The SBS synthesis technology was used to sequence the cDNA library using the Illumina HiSeq Xten high-throughput sequencing platform. The raw data were filtered to remove spliced sequences and low-quality reads to obtain high-quality clean reads for subsequent analysis. Contigs, transcripts, and unigenes were obtained were obtained by assembling clean reads using the Trinity software. To functionally annotate unigenes for subsequent analysis, the assembled unigenes were systematically compared with several public databases: Clusters of Orthologous Groups (COG), Gene Onotology (GO), Kyoto Encyclopedia of Genes and Genomes (KEGG), Eukaryotic Orthologous Groups (KOG), Protein Family Database (Pfam), Swiss-Prot Protein Sequence Database (Swiss-Prot), Evolutionary Genealogy of Genes: Non-supervised Orthologous Groups (eggNOG), and Non-Redundant Database (Nr). Genes with Log_2_FC > 1 and FDR < 0.05 were screened as differentially expressed genes (DEGs).

### Quantitative real-time PCR

Quantitative real-time PCR (qRT-PCR) was performed to validate the results of RNA-seq. Total RNA was extracted from petals at four times as previously described. First-strand cDNA was synthesized from total RNA according to the instructions of FastKing RT Kit with gDNA (KR116, Tiangen, China). qPCR was performed with the following reaction parameters: 94 °C for 20 s, 40 cycles of 94 °C for 10 s and 60 °C for 20 s, and 60 °C for 30 s. A 10 μL reaction system was used as follows: 1 μL cDNA template, 5 μL qPCR Master Mix (Lanyun, China), 0.2 μL forward primer, 0.2 μL reverse primer, and 3.2 μL nuclease-free water.

The *Actb* gene (Beta-actin7) was employed as the reference gene for normalization^39^. The quantification of qRT-PCR expression of nine selected differential genes obtained by the RNA-seq. 2^−ΔΔCT^ method was used to normalise the relative expression of the DEGs^40^. All qRT-PCR experiments were repeated three times.

## Results

### Floral scent components and trends in fragrance release from petals of *C. nocturnum*

A volatile substances total ion chromatogram (TIC) was obtained from 18:00 (CK) to 06:00 of the next day by GC–MS analysis (Fig. 1C). The main components of the volatiles obtained by GC–MS analysis were essentially consistent from 18:00 to the next 04:00. A total of 22 well-matched compounds were detected in the volatiles from the petals of *C. nocturnum* (Table 1), including 12 species of phenylpropanoids.

**Table 1:** Volatile components in the petals of *C. nocturnum.*

Code	Retention time (min)	Volatile compound	Molecular formula	Mw
1	0.72	3-Hexen-1-ol (*E*)-	C_6_H_12_O	100.1589
2	1.421	1-Butanol, 3-methyl-, acetate	C_7_H_14_O_2_	130.18486
3	4.968	Benzaldehyde	C_7_H_6_O	106.12194
4	7.036	3-Hexen-1-ol, 1-acetate, (3*E*)-	C_8_H_14_O_2_	142.2
5	8.224	Benzyl alcohol	C_7_H_8_O	108.13782
6	8.658	Phenyl acetaldehyde	C_8_H_8_O	120.15
7	8.87	1,3,7-Octatriene, 3,7-dimethyl-	C_10_H_16_	136.23404
8	9.954	Benzoylformic acid	C_8_H_6_O_3_	150.13144
9	9.983	Ethanone, 2-(formyloxy)-1-phenyl-	C_9_H_8_O_3_	164.158
10	10.905	Methyl benzoate	C_8_H_8_O_2_	136.15
11	11.251	1,6-Octadien-3-ol, 3,7-dimethyl-	C_10_H_18_O	154.25
12	11.676	Phenylethyl alcohol	C_8_H_10_O	122.16
13	13.86	Benzyl acetate	C_9_H_10_O_2_	150.17
14	14.9	2-Butoxybenzoic acid methyl ester	C_12_H_16_O_3_	208.25364
15	16.958	1-Adamantanecarboxylic acid, 2-phenylethyl ester	C_10_H_12_O_2_	164.2
16	19.338	Methyl anthranilate	C_8_H_9_NO_2_	151.16
17	19.655	Eugenol	C_10_H_12_O_2_	164.2
18	22.657	3-Buten-2-one,4-(2,6,6-trimethyl-1-cyclohexen-1-yl)-	C_13_H_20_O	192.3
19	22.915	1,3,6,10-Dodecatetraene, 3,7,11-trimethyl-, (*Z*, *E*)-	C_15_H_24_	204.351
20	23.207	1,3,6,10-Dodecatetraene, 3,7,11-11-trimethyl-	C_15_H_24_	204.351
21	24.553	Furan, 3- (4,8-dimethyl-3,7-nonadienyl)-, (*E*)-	C_15_H_22_O	218.3346
22	24.637	7,11-Dimethyldodeca-2,6,10-trien-l-ol	C_30_H_50_	410.72

The total peak area of the volatile substances was greatest at 24:00 and smallest at 06:00, with a 13.97-fold difference (Fig. 1B-a). Phenylpropanoids had maximum peak areas at 24:00 (Fig. 1B-b). At this time, the relative peak areas of benzaldehyde, phenylacetaldehyde, and benzyl acetate were 32.40%, 26.91%, and 23.07%, respectively. These three substances were the main components of phenylpropanoids substances. When the peak area of terpenoids attained maximum at 22:00 (Fig. 1B-c), 1,3,6,10-Dodecatetraene, 3,7,11-11-trimethyl- (5.25%), 1,6-Octadien-3-ol, 3,7-dimethyl- (0.25%) and Furan, 3- (4,8-dimethyl-3,7-nonadienyl)-, (*E*)- (0.21%) were the most abundant. The maximum peak area of fatty acid derivatives was at 04:00 (Fig. 1B-d). The relative peak areas of 3-Hexen-1-ol,1-acetate, (3*E*)- and 3-hexen-1-ol were 0.42% and 0.20%, respectively.

### Transcriptional analysis of genes during fragrance release

A total of 92.40 GB of clean data was obtained. The Q30 percentage was 91.65% and the CG percentages was above 42.43%, which indicated applicability that could be further analysed. 209,966 transcripts and 73,100 unigenes were assembled by reads, with N50 values of 1790 and 1851 bp, respectively, which reflected a high-quality assembly. Of all the unigenes, 288,876 were longer than 1000 bp, which represented 39.5% of the total (Table S1). The Pearson correlation coefficient (R^2^) was used to assess the correlation between samples; the closer R^2^ was to 1, the stronger the correlation between the two samples. The R^2^ between biological replicate samples within the same group were all greater than 0.849, demonstrating high reproducibility and supporting their suitability for further analysis (Fig. S1).

**Fig. 2:**
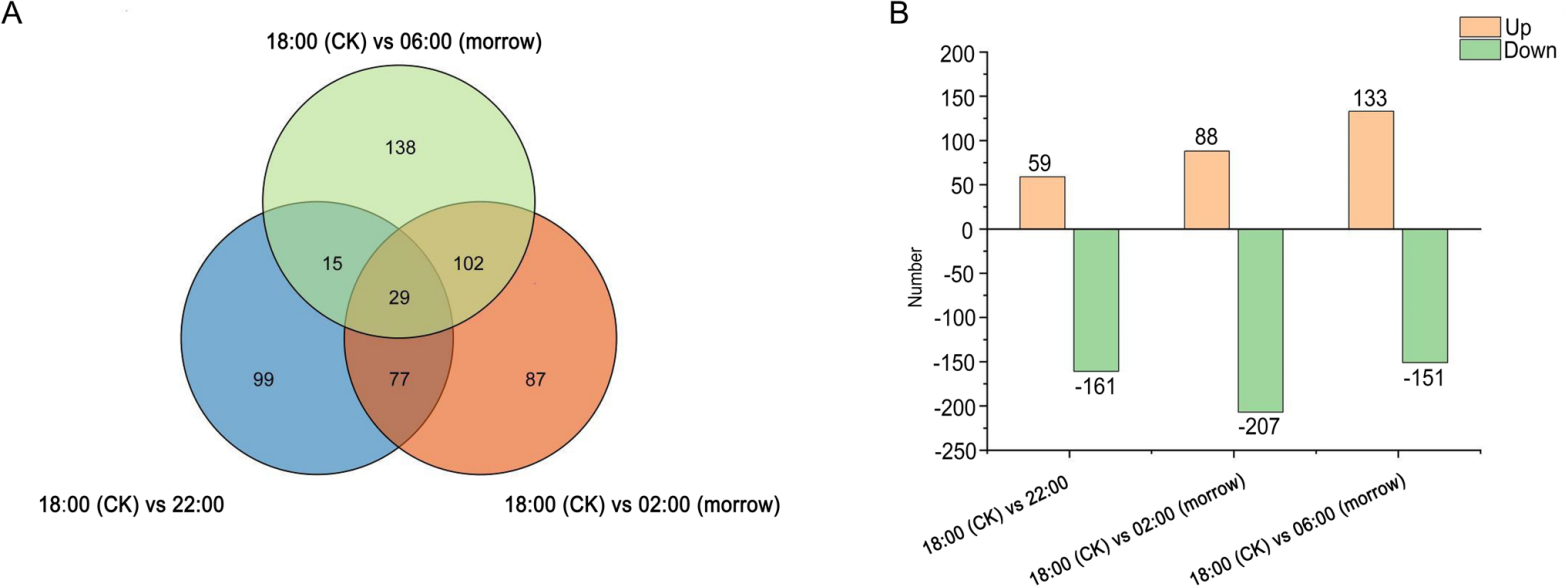
Analysis of DEGs in the petals of *C. nocturnum*. (**A**) Venn diagram of DEGs; blue, orange and green circles indicate the number of DEGs at 18:00 (CK) versus 22:00, 18:00 (CK) versus 02:00 (morrow) and 18:00 (CK) versus 06:00 (morrow), respectively; overlap of colours indicates the number of shared DEGs. (**B**) Statistical bar chart of the number of DEGs; orange and green indicate up- and downregulated DEGs, respectively.

Comparison of the four periods revealed a total of 799 differentially expressed genes at Log_2_FC > 1 and FDR < 0.05. Of these DEGs, 280 (35.04%) were upregulated and 519 (64.96%) were downregulated. There were significant differences in gene expression at different flowering stages (Fig. 2A,B). Compared to the 18:00 control, 02:00 had the most DEGs, with 88 upregulated and 207 downregulated genes. There were 59 upregulated and 161 downregulated genes at 22:00. At 06:00 there were 284 DEGs, comprising 133 upregulated and 151 downregulated genes. These up- and downregulated genes were important for revealing key regulatory genes associated with biological activities in *C. nocturnum*.

#### Enrichment analysis of differentially expressed genes

To obtain more information about the unigenes, the entire set of unigene sequences was subjected to a BLAST (Basic Local Alignment Search Tool) search against database. A total of 728 unigenes were functionally annotated from 799 DEGs. Among them, 196 unigenes were annotated at 18:00 (CK) versus 22:00, 267 unigenes were annotated at 18:00 (CK) versus 02:00, and 265 unigenes were annotated at 18:00 (CK) versus 06:00. Comparison of the four periods revealed that 588 and 689 unigenes were annotated to Pfam and eggNOG, respectively, while 578 and 728 unigenes were annotated to Swiss-Prot and Nr, respectively (Table S2).

**Fig. 3:**
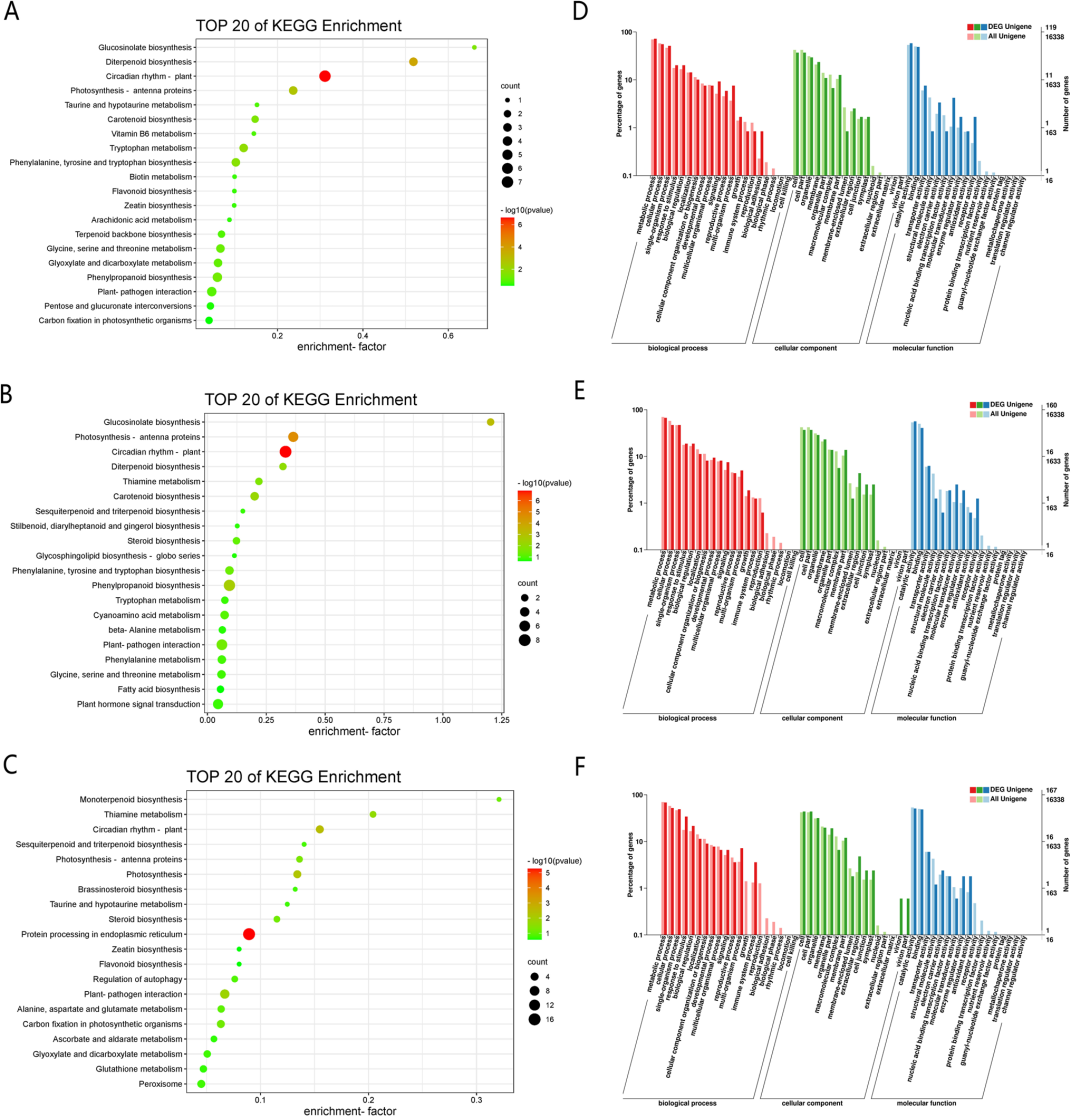
KEGG pathway enrichment and GO enrichment analysis of differentially expressed genes. KEGG enrichment analysis at (**A**) 18:00 (CK) versus 22:00, (**B**) 18:00 (CK) versus 02:00 (morrow) (**C**) 18:00 (CK) versus 06:00 (morrow). GO enrichment analysis at (**D**) 18:00 (CK) versus 22:00 (**E**) 18:00 (CK) versus 02:00 (morrow) (**F**) 18:00 (CK) versus 06:00 (morrow).

GO significant enrichment analysis showed that 446. Using 18:00 as the control, 119, 160, and 167 DEGs were annotated at 22:00, 02:00, and 06:00, respectively. DEGs were grouped into three main categories: biological processes, molecular functions, and cellular components (Table S2). 79, 94, and 104 DEGs were annotated with metabolic processes (GO:0008152), 58, 72, and 79 DEGs with single-organism processes (GO:0044699), 40, 51, and 85 DEGs with catalytic activities (GO:0003824), 69, 90, and 69 DEGs with intracellular processes (GO:0005622), and 58, 65, and 81 DEGs with binding (GO:0005488), respectively (Fig. 3D–F).

The total number of DEGs annotated to the COG database in *C. Nocturnum* samples was 287 (Table S2). At 18:00 (CK) versus 22:00, 18:00 (CK) versus 02:00, and 18:00 (CK) versus 06:00, there were 70, 92, and 125 DEGs annotated, respectively. The statistical results of the COG classification of DEGs between sample groups were shown in Fig. S2A–C. The highest number of DEGs related to amino acid transport and metabolism, secondary metabolite biosynthesis and catabolism were observed at 22:00. The number of DEGs related to post-translational modifications, protein turnover and chaperone first decreased and then increased significantly from 18:00 (CK) to 06:00, peaking at 06:00.

The top 20 pathways in the KEGG enrichment pathway analysis were shown in Fig. 3A–C. The number of DEGs annotated to the KEGG database was 305. 76, 111 and 118 DEGs were annotated to 22:00, 02:00 and 06:00, respectively, with 18:00 as the control (Table S2). The main enriched pathways were circadian rhythm—plant (ko04712), photosynthesis-antenna proteins (ko00196), diterpenoid biosynthesis (ko00904), phenylalanine, tyrosine and tryptophan biosynthesis (ko00400), phenylpropanoid biosynthesis (ko00940), terpenoid backbone biosynthesis (ko00900) and plant hormone signal transduction (ko04075). These annotation results provide a valuable resource for gene identification and functional analysis during flower development in *C. nocturnum*. Among these, the phenylpropanoid biosynthesis pathway emerged as the most significant pathway regulating the synthesis of floral scent metabolites. Day–night alternates and plant hormones play important roles in the process of flowering.

#### Analysis of differentially expressed genes

Based on the transcriptome data, key pathways and annotated DEGs during flowering and fragrance release of *C. nocturnum* were analyzed. In addition, DEGs in the plant flowering pathway and the phenylpropanoid biosynthesis pathway were shown.

##### Plant flowering pathway

**Fig. 4:**
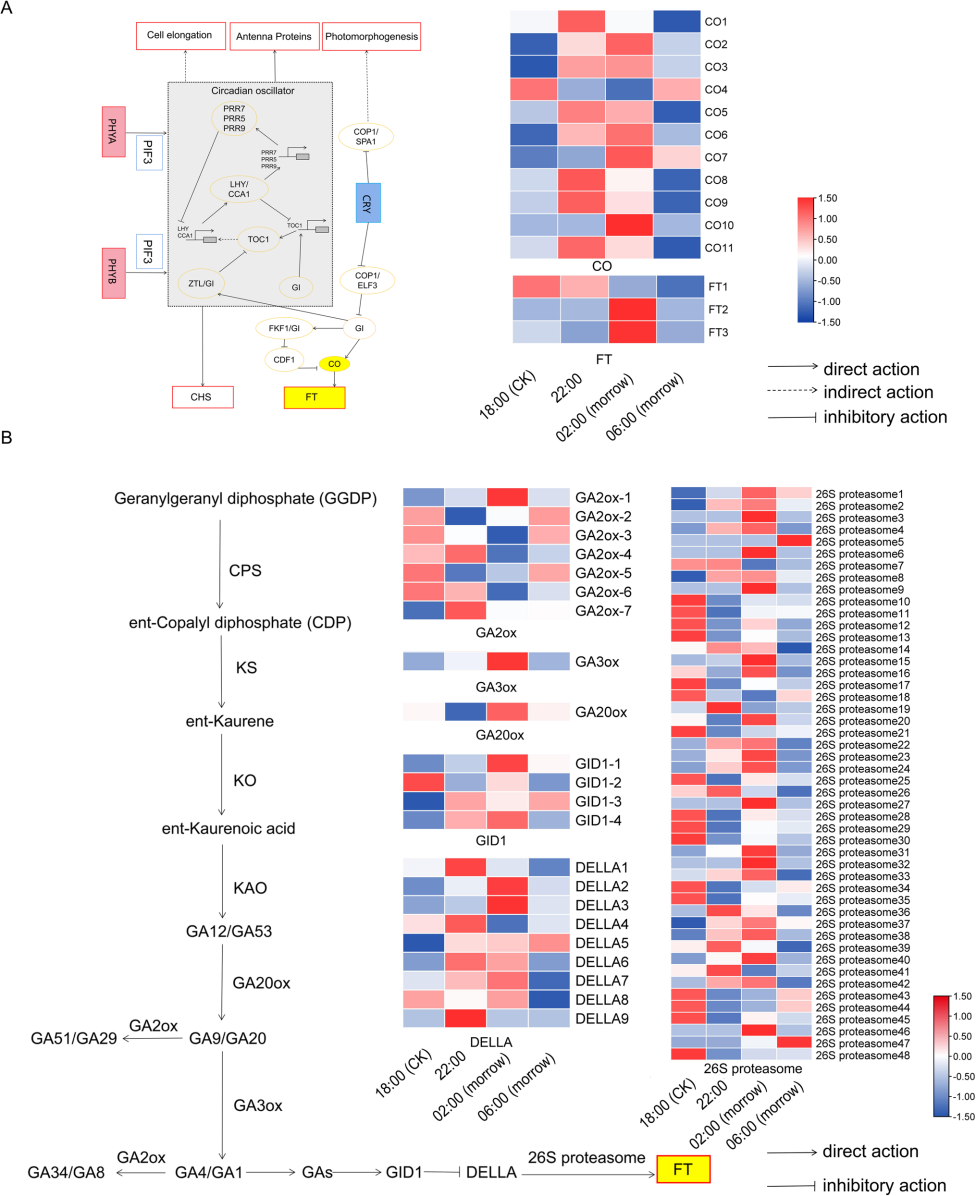
Heat map of differentially expressed genes encoding key enzymes in the flowering pathway. (**A**) Heatmap of key differentially expressed genes associated with day and night alternation. (**B**) Heatmap of gibberellin synthesis and signal transduction pathway and expression of its key genes.

Photosensitive pigment A (PHYA), photosensitive pigment B (PHYB) and cryptochrome (CRY) directly or indirectly regulate the expression of *CO*. *FT* is promoted by *CO*, thereby inducing flowering (Fig. 4A)^41^. The RNA-Seq expression profile showed that the expression level of *CO*s increased significantly from 18:00 to 22:00, and the 10 *CO*s were positively correlated with flowering time. *CO1*, *CO5*, *CO8* and *CO11* were significantly upregulated at 18:00 versus 22:00, with fold changes (FC) of 5.32, 5.28, 6.16 and 4.40, respectively. The expression levels of *FT2* (FC: 241.17) and *FT3* (FC: 6.06) were relatively higher at 02:00 than18:00.

**Fig. 5:**
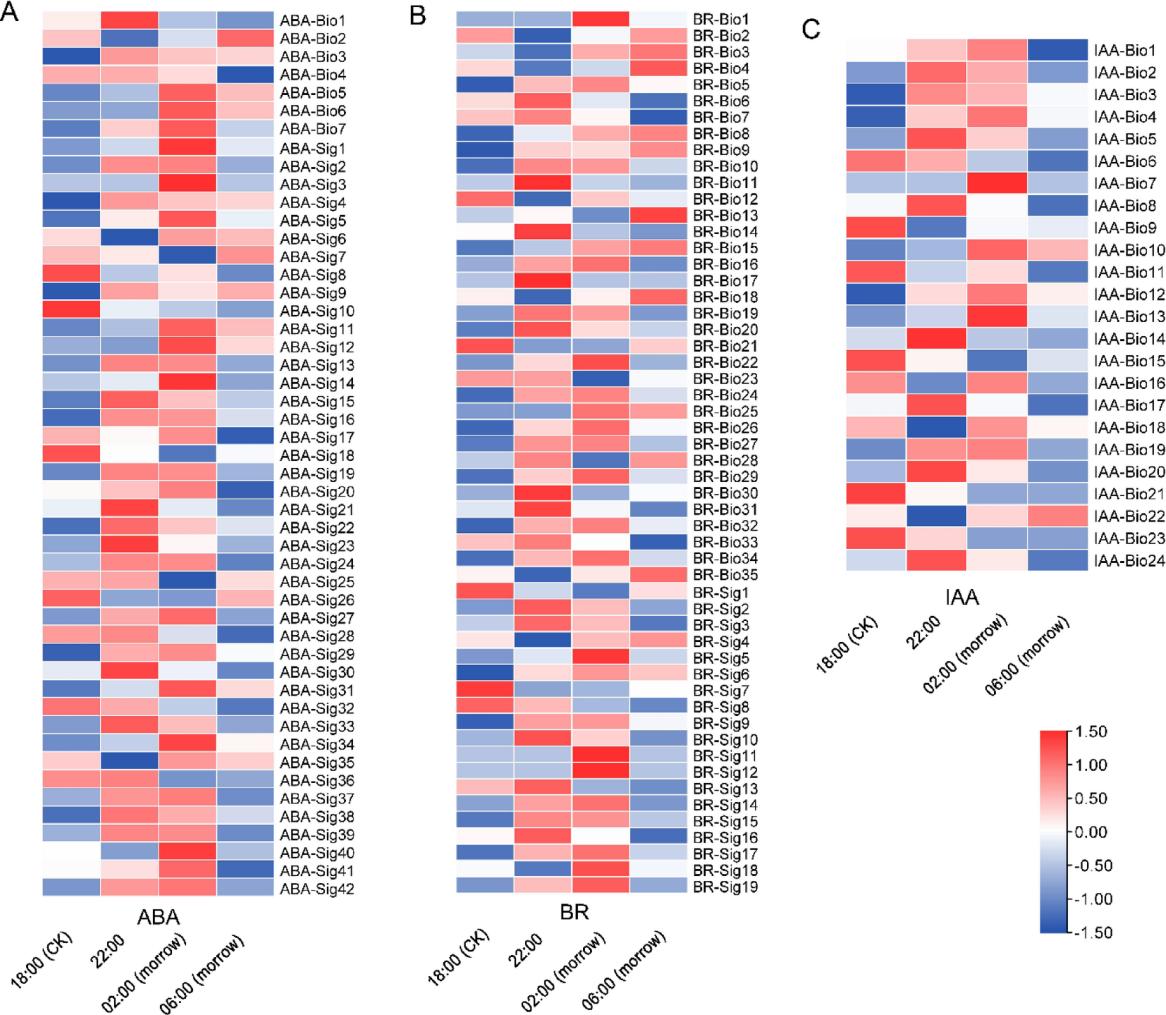
Heatmap of plant hormone biosynthesis and signalling associated with flowering.

The hormones related to plant flowering included GA, AB, BR and IAA, which may promote flowering under certain conditions^34,42^. The differential expression of key genes in this pathway was analysed. In the GA synthesis and signal transduction pathway (Fig. 4B), GA1 and GA4 are biologically active. GAs reduces the activity of DELLA inhibitory proteins by forming a complex with GID and DELLA, which is degraded by the action of 26S protease^43–45^. The next step is the promotion of *FT* expression, which in turn promotes plant flowering^46^. *GA 20 oxidase* (*GA20ox*) and *GA 3-oxidase* (*GA3ox*) had the highest expression levels at 02:00. *GA2ox-2*, *GA2ox-3*, *GA2ox-5* and *GA2ox-6* showed the opposite trend, with low expression levels at night*. GID1-1* and *GID1-4* were upregulated at 18:00 (CK) versus 02:00. Addition, 26 genes encoding *26S proteasome* were positively correlated with flowering time. A total of 49, 54 and 33 genes related to ABA, BR and IAA synthesis and signal transduction were detected, respectively, including 40, 43 and 18 genes that were positively correlated with the flowering time. This meant that the trend in the expression levels of most genes was upregulation followed by downregulation (Fig. 5).

##### Phenylpropanoid biosynthesis pathway

**Fig. 6:**
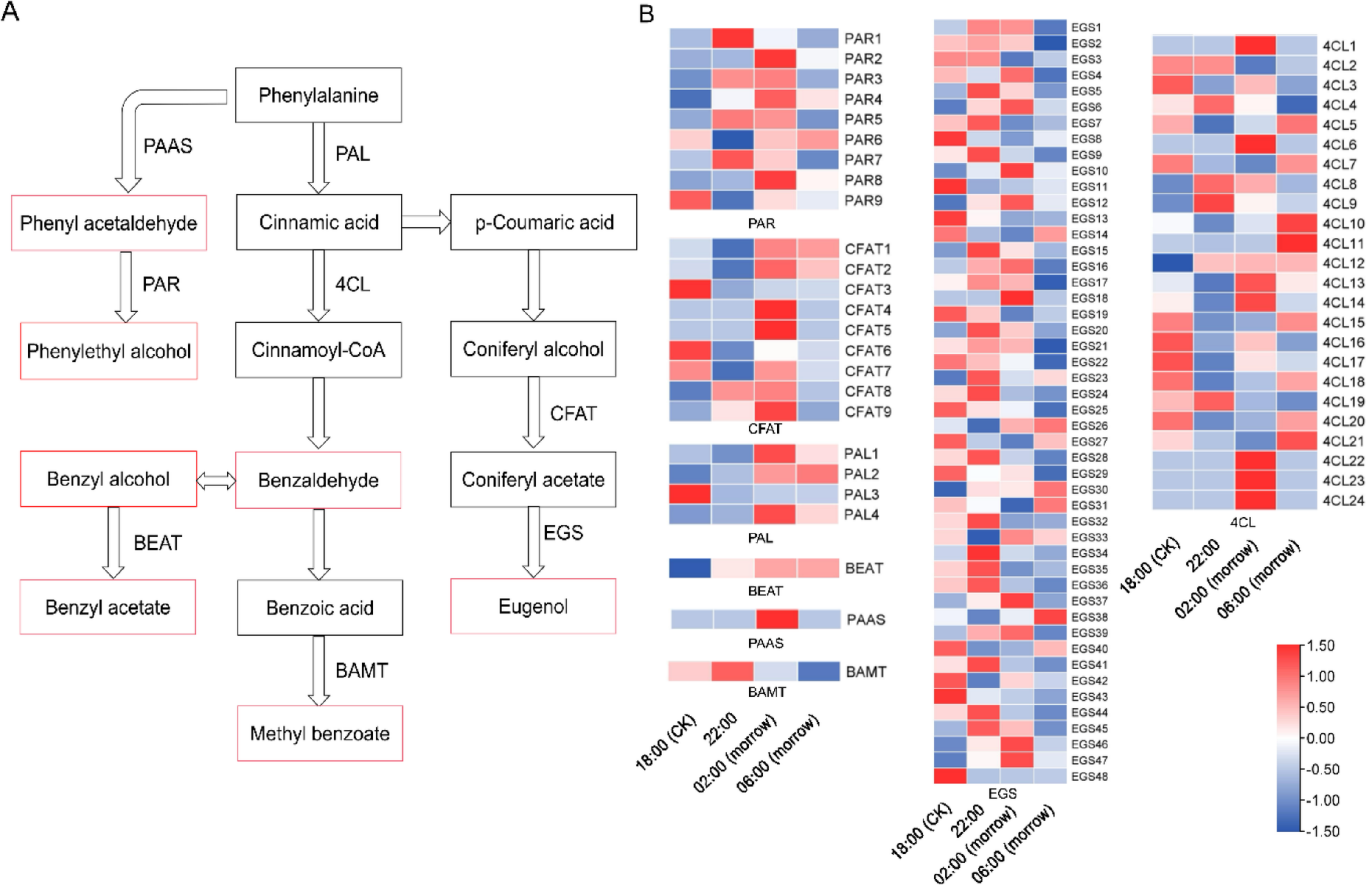
Heat map of DEGs in the phenylpropanoid biosynthesis pathway. The red rectangles indicate the metabolites that were detected. The arrows indicate the synthesis steps.

The phenylpropanoid biosynthesis pathway contains the seven key floral scent components of *C. nocturnum*: benzaldehyde, phenyl acetaldehyde, phenylethyl alcohol, benzyl alcohol, benzyl acetate, methyl benzoate and eugenol. To identify crucial genes affecting floral scent synthesis, DEGs in the phenylpropanoid biosynthesis pathway were analysed (Fig. 6A), including *PAL*, *4CL*, *PAAS*, *PAR*, *BEAT*, *BAMT*, *CFAT* and *EGS* (Fig. 6B)*.* There was significantly higher activity of *PAL1* and *PAL4* at 02:00 than at 18:00. *PAL4* expression level at 02:00 was 2.70-fold higher than at 18:00. Fourteen genes encoding *4CLs* and *BEAT* were upregulated in 18:00 versus 22:00. Eighteen DEGs encoding *PARs* and *CFATs* were identified, most of which were highly expressed from 22:00 to 02:00 at night. Additionally, the expression levels of 33 *EGSs* were much higher at 22:00 and 02:00 than at the other two times and were positively correlated with floral scent release.

#### Quantitative analysis by qRT-PCR

**Fig. 7:**
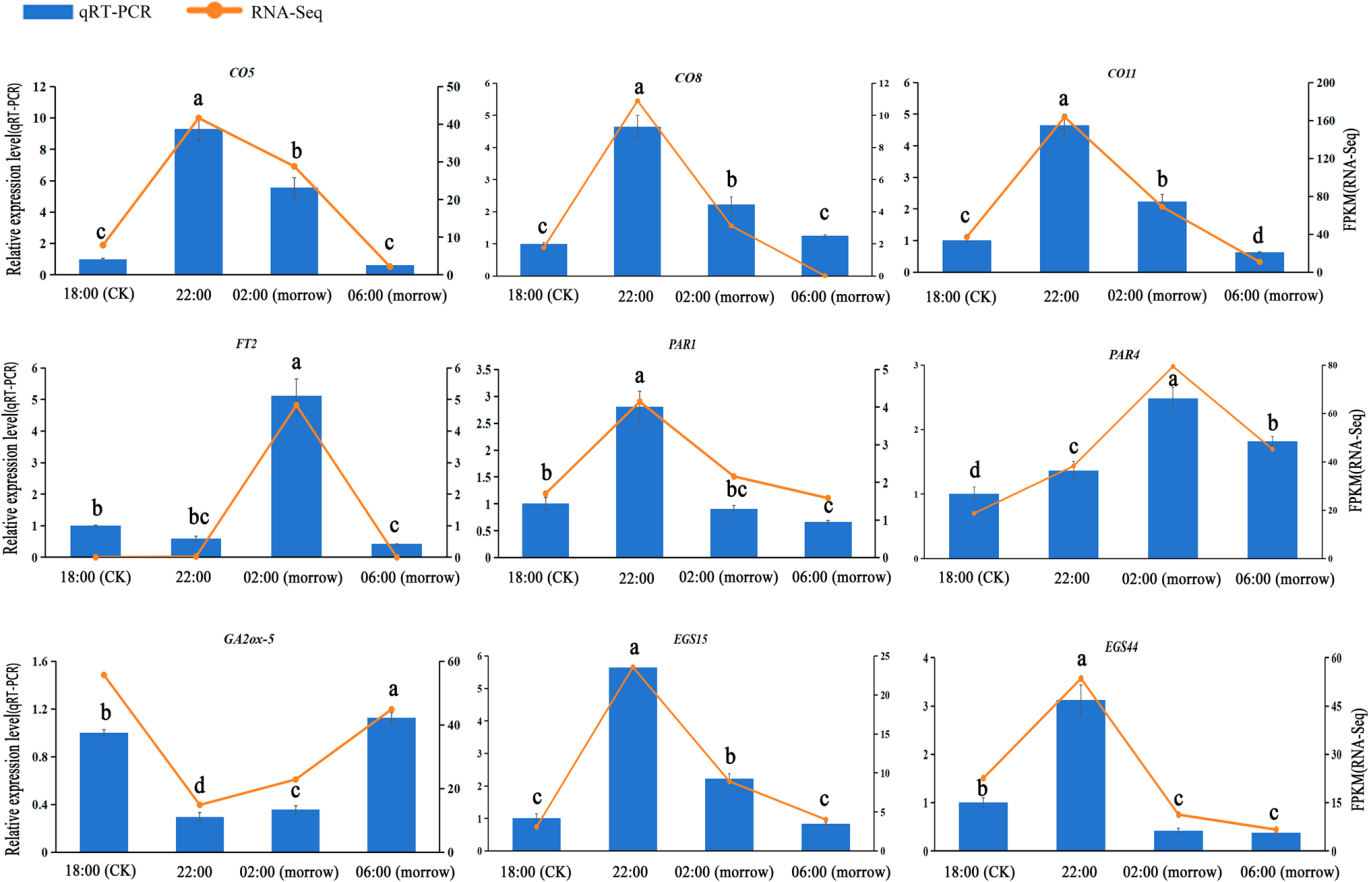
Quantification of qRT‒PCR expression of nine selected genes obtained by RNA-seq. Different letters (a, b, c, d) indicate significant differences at different sampling times (*p* < 0.05).

To validate the results of the transcriptome genetic analysis, qPCR was performed for nine representative genes with significant expression differences (Fig. 7). These included four genes (*PAR1*, *PAR4*, *EGS15*, *EGS44*) from the phenylpropanoid biosynthesis pathway related to floral scent synthesis, four genes (*CO5*, *CO8*, *CO11*, *FT2*) related to flowering and one hormone synthesis gene (*GA2ox-5*). The expression of *PAR*s, *EGSs*, *CO*s and *FT*s tended to increase sharply and then decrease, and these genes were highly expressed at 22:00 or 02:00. *GA2ox-5* expression showed the opposite trend of decreasing and then increasing, with the lowest expression at 22:00. The qRT-PCR results of the DEGs showed the same trend as the RNA-Seq results, indicating the high reliability of the data obtained by transcriptome sequencing.

## Discussion

**Fig. 8:**
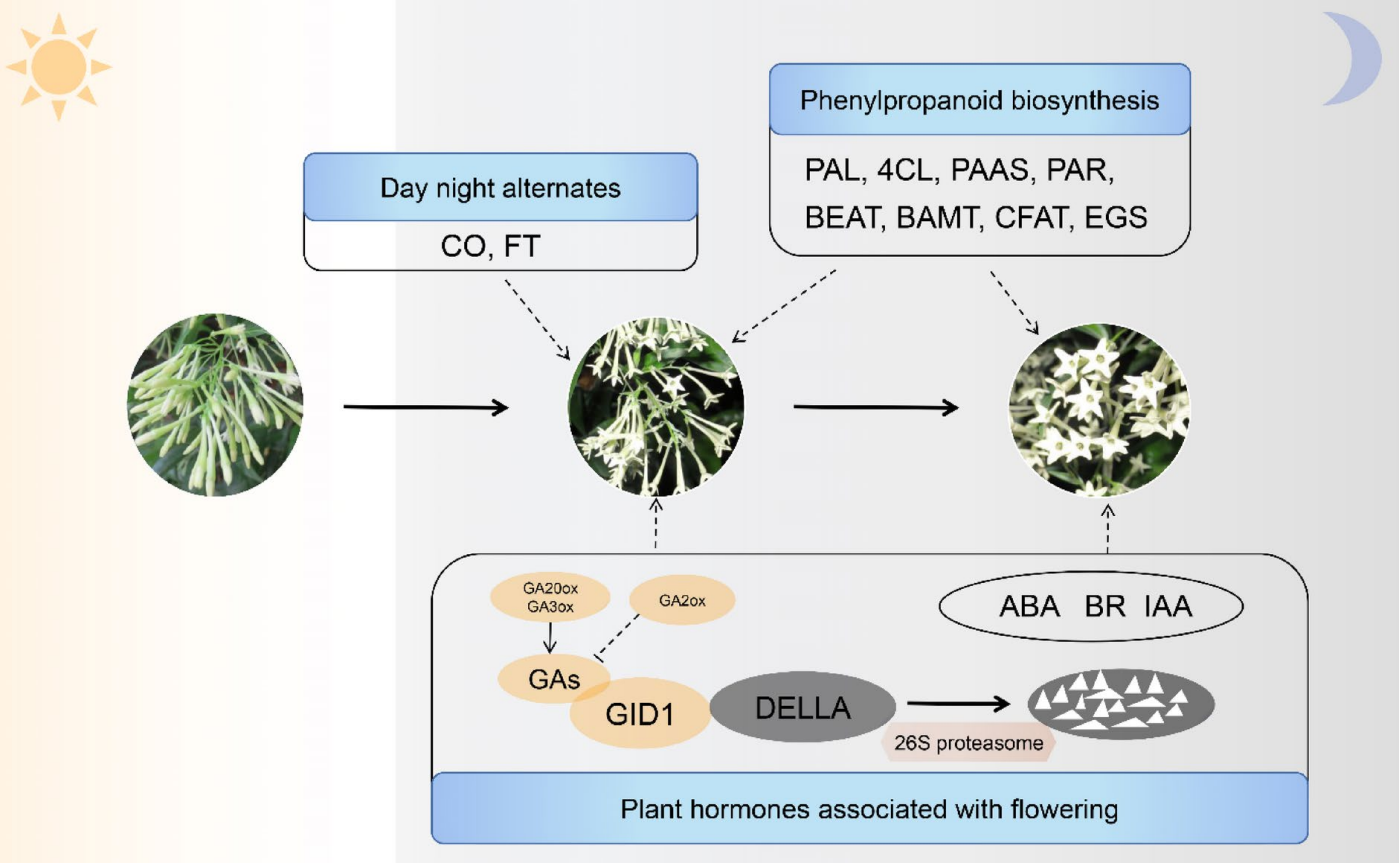
Diagram of the flowering and fragrance release mechanism of *C. nocturnum*.

The expression levels of DEGs in the phenylpropanoid biosynthesis pathway during the flowering stage were significantly increased at 22:00 or 02:00. The same trend was observed for genes related to day–night alternates and plant hormones. These genes may be directly responsible for the flowering and fragrance release of *C. nocturnum* (Fig. 8).

The peak areas of phenylpropanoids followed the same trend as the total peak area in this study, both being highest at 24:00 and lowest at 06:00. When the peak area of phenylpropanoids attained maximum at 24:00, benzaldehyde, phenylacetaldehyde, and phenylmethyl acetate emerged as dominant components, accounting for 32.40%, 26.91%, and 23.07%, respectively. As a result, the largest proportions of benzaldehyde, phenylacetaldehyde and phenylmethyl acetate were detected during the night when the aroma was strong. These components were identified as the main volatile substances in *C. nocturnum*. Phenylpropanoid biosynthetic pathways in flowers such as *Prunus mume*^46^ and *Ipomoea nil*^47^ have been studied in more detail. *PAL*, which is closely related to the synthesis of phenylpropanoid substances, is highly expressed in *Petunia hybrida*^26^ during the blooming stage. The expression of *BEAT* in *Prunus mume* is higher during the flowering stage than other stages and promotes the production of floral scent substances^48^. *RcEGS1* is highly expressed during the bloom period in *Rosa chinensis*^49^. *RfBAMT* plays a crucial role in the formation and regulation of methyl benzoates in *Rhododendron fortune*^50^. The results of the present study showed that the phenylpropanoid biosynthesis pathway is important for floral scent release by *C. nocturnum*. *PAL*s, *PAR*s, *BPBT*, *4CL*s, *CFAT*s, *BAMT*, *BEAT* and *EGS*s were highly expressed from 22:00 to the next 02:00, suggesting that these genes may positively regulate the synthesis of phenylpropanoids. These genes may play a significant role in regulating fragrance release in *C. nocturnum*.

Aroma release accompanies plant flowering. *CO* is a major transcription factor in the flowering regulatory process and can activate *FT* expression^51^. *CO*-*FT* plays a key regulatory role in the induction of flowering in *Populus trichocarpa*^52^. *AhFT-21 and AhFT-22* of *Arachis hypogaea* are highly expressed during the flowering stage, which may be closely related to its flowering regulation^51^. In the present study, *CO* and *FT* showed an overall increasing trend in expression accompanying the flowering process of *C. nocturnum*. This interpretation was supported by previous studies in which *CO* activated *FT* expression levels and positively regulated flowering. Here, it is hypothesised that *CO* and *FT* are key genes for nighttime and daytime flowering; however, the periods in which these genes function may be different. The pathways by which the *CO* and *FT* genes function need to be further investigated.

Plant hormones are important for regulating flowering and floral release^33^. GAs interact with other hormone signalling pathways, such as IAA and BR, to regulate plant flowering^32,53,54^. *GA2ox* reduces GA activity in plants^55^. Overexpression of *JcGA2ox7* or *JcGA2ox8* reduces the level of active GAs, leading to delayed flowering in *Arabidopsis thaliana*^56^. *SbGA20ox1* and *SbGA20ox3* positively regulate GAs in *Sorghum*^57^*. TaGA3ox2* positively regulates in *Triticum aestivum*^58^. Here, it is speculated that *GA3ox*s and *GA20ox*s are highly expressed at night, while *GA2ox*s shows the opposite trend. The high expression levels of *GA20ox*s and *GA3ox*s may promote the synthesis of active GAs. In contrast, low expression levels of *GA2ox*s may indirectly leads to the accumulation of active GAs. Trends in the expression levels of genes related to ABA, IAA and BR synthesis were consistent with the flowering time.

## Conclusion

The detection of floral fragrance substances in *C. nocturnum* showed that benzaldehyde, phenyl acetaldehyde and benzyl acetate were the main components. *PALs*, *PAAS*, *PARs*, *4CLs*, *CFATs*, *BEAT*, *EGSs* and *BAMT* genes followed the same trend as fragrance release time. *CO*, *FT* and genes related to phytohormone (GA, IAA, BR, ABA) synthesis and signal transduction were positively correlated with the flowering of *C. nocturnum*. These genes may be important differential genes that regulate the fragrance release and flowering. These results deepen understanding of the mechanism of flowering and fragrance release in *C. nocturnum*, laying the foundation for further studies as well as providing theoretical support for the study of other aromatic plants.

## Electronic supplementary material

Below is the link to the electronic supplementary material.

Supplementary Material 1

Supplementary Material 2

## Data Availability

Data availability statement: Sequence data that support the findings of this study have been deposited in the NCBI database with the primary accession code PRJNA1203082 (http://www.ncbi.nlm.nih.gov/bioproject/120382).
