# Supplementary material for: Unraveling the mechanism of fragrance release in Cestrum nocturnum through transcriptome and volatile compound profiling
Source: Sci Rep. 2025 May 2;15:15376. doi: 10.1038/s41598-025-99542-3 (PMC12048523; doi:10.1038/s41598-025-99542-3)
Supplement: Supplementary file 1 — Supplementary Material 1 [file 41598_2025_99542_MOESM1_ESM.docx]

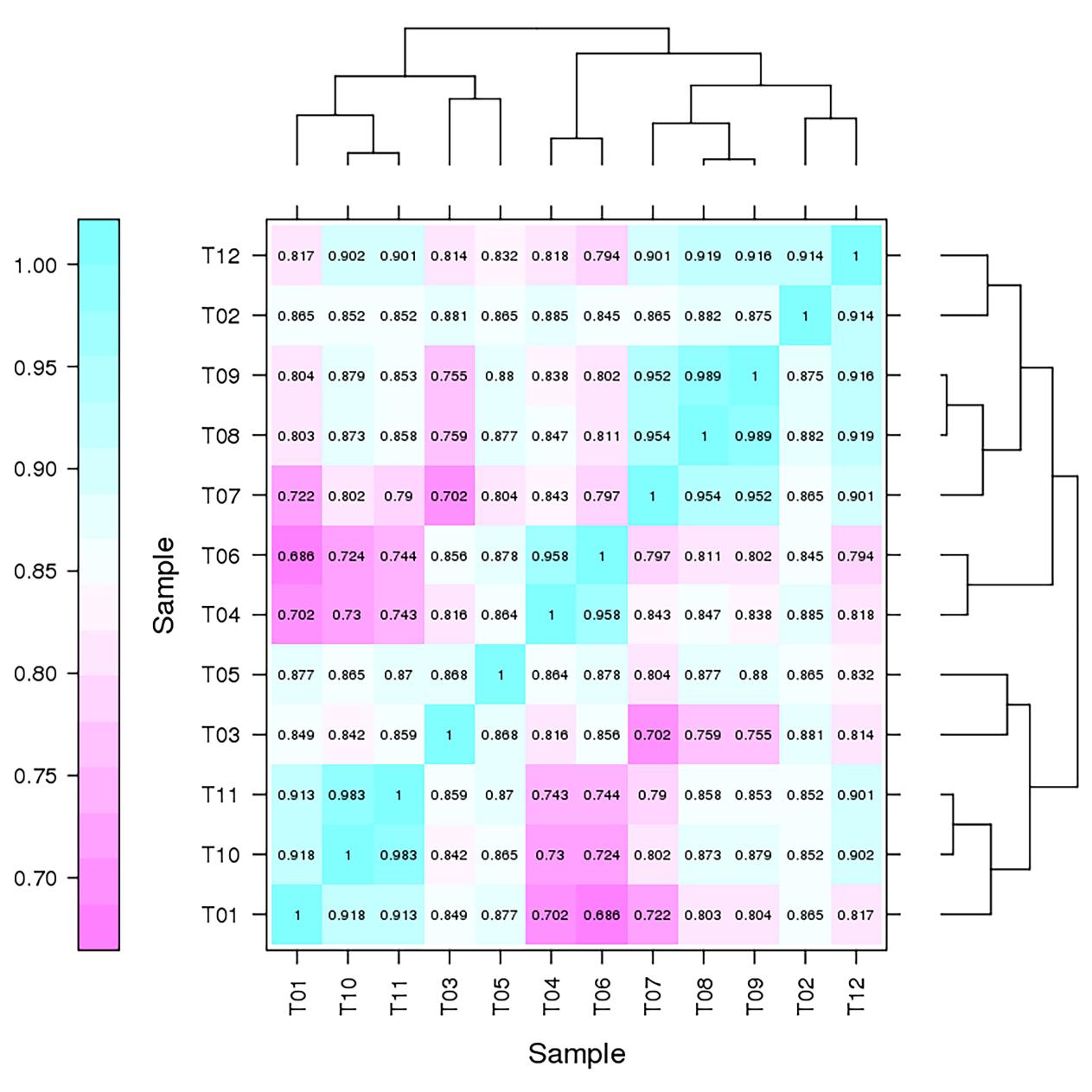


**Figure S1. Correlation heat map of samples**

The squares of Pearson correlation coefficients between each two samples were showed in the array.


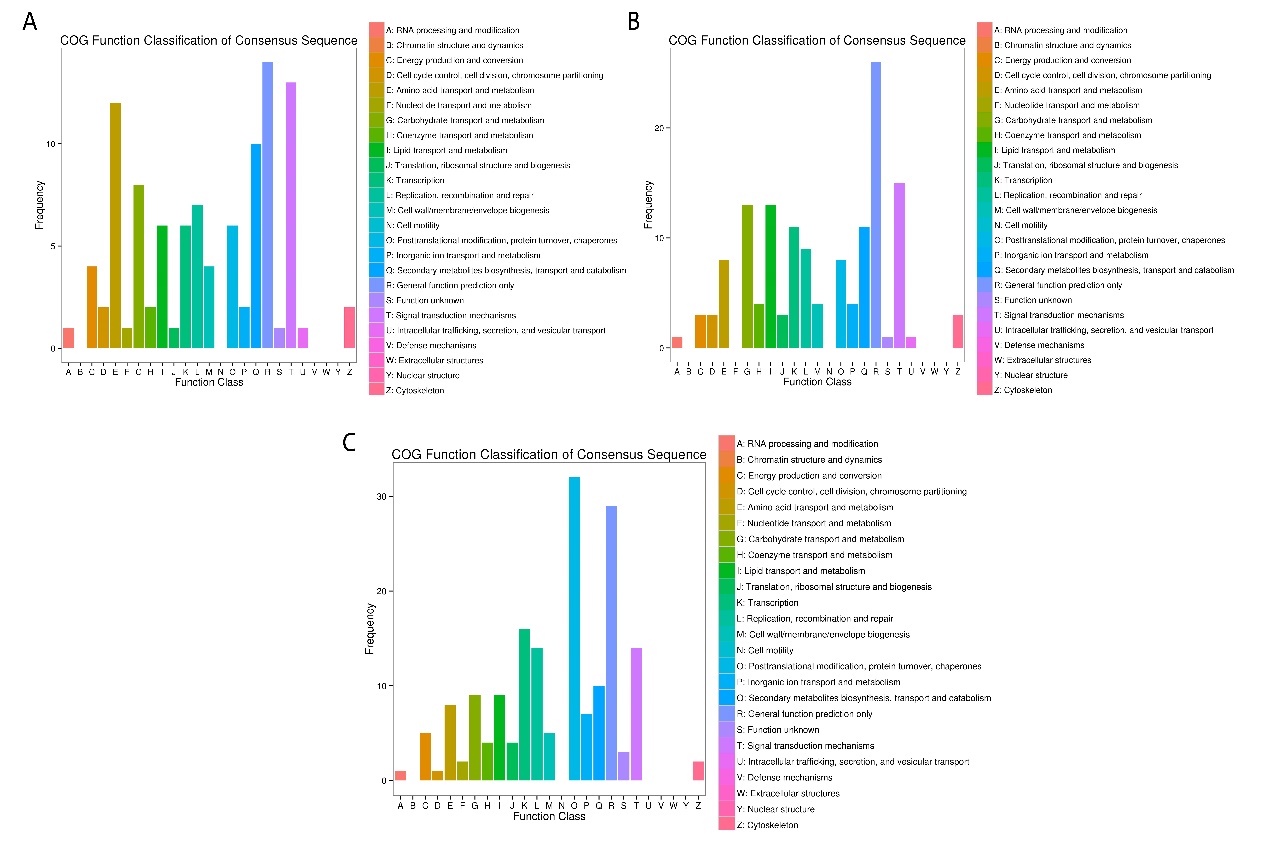


**Figure S2. COG annotation classification of differentially expressed genes.**

COG annotation classification at (A)18:00 (CK) vs 22:00, (B)18:00 (CK) vs 02:00 (C)18:00 (CK) vs 06:00
